# Beyond the acoustic diffraction limit: superresolution localization optoacoustic tomography (LOT)

**DOI:** 10.1038/s41377-018-0029-6

**Published:** 2018-06-13

**Authors:** Chulhong Kim

**Affiliations:** Departments of Creative IT Engineering, Electrical Engineering, Mechanical Engineering, Interdisciplinary Bioscience and Bioengineering, Pohang University of Science and Technology, Pohang, Gyeongbuk 37673 Republic of Korea

**Localization optoacoustic tomography provides superresolution imaging capability in 3D beyond the acoustic diffraction limit, which can be crucial for mapping microcirculation in cancers, brain functions, peripheral vascular diseases, etc.**

Optoacoustic (also referred to as photoacoustic) tomography (OAT) has been gaining popularity for preclinical and clinical imaging during the past couple of decades^1^. OAT breaks the long-standing shallow imaging depth limitation of conventional optical imaging by forming an image using the optoacoustic (OA) effect. Through advances in ultrasound imaging technologies, OAT provides rich optical contrast while achieving high spatial resolution deep inside living subjects (up to several centimeters). Thanks to these hybrid technologies, the use of preclinical OAT to study cancer physiopathology, neural physiology, drug delivery, vascular diseases, etc., has spread globally to many laboratories. More importantly, the applications of OAT have been extended to include many clinical trials, such as early diagnosis and treatment monitoring of cancers, imaging of the bowel for diseases, human neuroimaging for diagnosing neurological defects, imaging of peripheral arteries and veins for detecting vascular disease, and intravascular imaging for characterizing plaque.

OAT is mainly implemented in two domains: the optical ballistic regime (<~1 mm in biological tissues) and the optical diffusive regime (>~1 mm in biological tissues). In the optical ballistic regime, the lateral resolution of OA imaging is determined by the tight optical focus, and the technology is referred to as optical-resolution OA microscopy (OAM). In the optical diffusive regime, the resolution is determined by the acoustic focus and/or ultrasound parameters, and this technology is referred to as acoustic-resolution OAT. The resolutions of OAT in both regimes are limited by either the optical or acoustic diffraction limit. Recently, several attempts were made by multiple researchers to exceed these diffraction limits to achieve superresolution imaging. In the optical ballistic domain, Lihong’s group explored subdiffraction OAM using either nonlinear optical saturation or photobleaching effects^2, 3^. In addition, Lee et al. developed superresolution photoactivated atomic force microscopy and improved the resolution to ~8 nm^4^. In the optical diffusive domain, Thomas et al. demonstrated superresolution OAT beyond the acoustic diffraction limit by either probing the fluctuations of OA signals with dynamic optical speckle excitation or detecting the fluctuating OA signals from flowing optical absorbers^5, 6^. Furthermore, Donald et al. applied a wavefront-shaping technology to squeeze the spatial resolution of OAT to smaller than the acoustic diffraction limit ^7^.

**Fig. 1:**
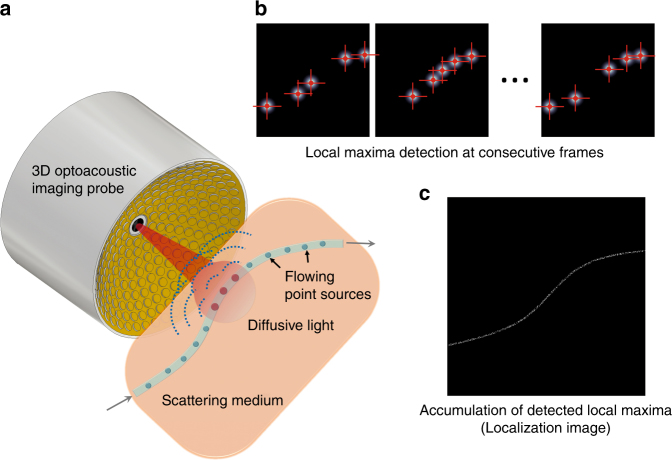
Imaging principles of localization optoacoustic tomography. **a** A spherical array of ultrasound transducers is used to acquire a three-dimensional optoacoustic image of flowing absorbers in an optical scattering medium for each laser pulse. **b** The positions of sparsely distributed absorbers are measured (localized) in a sequence of images. **c** An image is formed by superimposing the localized positions

Compared to the previous results, Luís et al. applied the localization imaging approach to OAT to enhance the visualization of flowing particles that are embedded in an optical scattering medium in 3D and referred to this approach as localization optoacoustic tomography (LOT). In LOT, multiple OA images are obtained at the same location using a portable volumetric OAT system that is equipped with a spherical array probe (Fig. 1a)^8^. Then, the OA images are superimposed after filtering each image to obtain local maximum pixels (bright dots in Fig. 1b) that correspond to the particle’s OA signals. In the final superimposed image, the OA signals overlap and the final LOT image shows the path of the moving particle in 3D (Fig. 1c). Compared to the conventional OA images, the blurring effect of the point spread function disappears in the LOT images since only the locations of the point sources are used for localization of the signals. For high-resolution imaging of vasculatures, injection of exogenous agents into the blood stream is required. Table 1 compares the mechanisms and performances from previous reports with those of LOT.

**Table 1:** Comparison of the imaging domain, superresolution mechanism, image formation mechanism, and dimensions from previous reports and of LOT

	Imaging domain	Superresolution mechanism	Image formation mechanism	Dimensions
Lee et al.^4^	Surface	OA/photothermal detection using AFM tips	Point-by-point scanning	2D in *x* and *y*
Danielli et al.^2^	Ballistic	Nonlinear optical excitation using optical saturation	Point-by-point scanning	3D
Yao et al.^3^		Nonlinear optical excitation using photobleaching		
Chaigne et al.^5, 6^	Diffusive	Fluctuations of OA signals with dynamic optical speckle excitation	Ultrasound beamforming using a linear array probe	2D in *x* and *z*
		Fluctuations of OA signals with flowing particles		
Conkey et al.^7^		Optical excitation with wavefront shaping	Point-by-point scanning	3D
Luís et al.^8^		Fluctuations of OA signals with flowing particles	Ultrasound beamforming using a spherical array probe	3D

LOT is suitable for imaging any structure with the flow of optical absorbers. For biomedical applications, imaging of vascular structures with high resolution is very attractive for studying cancers, brain activities, peripheral vascular diseases, etc.
